# P-1205. The Impact of the COVID-19 Pandemic on Hospitalizations Associated with Respiratory Syncytial Virus (RSV) Illness Among Children and Adolescents in Ontario, Canada

**DOI:** 10.1093/ofid/ofae631.1387

**Published:** 2025-01-29

**Authors:** Sazini Nzula, Alexandra Goyette, Deshayne B Fell, Ceryl Tan, Natalie Nightingale, Maria Esther Perez Trejo, Calum S Neish, Ana Gabriela Grajales

**Affiliations:** Pfizer Canada, Kirkland, Quebec, Canada; Pfizer Canada, Kirkland, Quebec, Canada; Pfizer Inc., New York City, New York; IQVIA Solutions Canada Inc, Mississauga, Ontario, Canada; IQVIA Solutions Canada Inc, Mississauga, Ontario, Canada; IQVIA Solutions Canada Inc, Mississauga, Ontario, Canada; IQVIA Solutions Canada Inc, Mississauga, Ontario, Canada; Pfizer Canada ULC, Kirkland, Quebec, Canada

## Abstract

**Background:**

COVID-19 pandemic measures may have lowered general immunity against respiratory syncytial virus (RSV). It is therefore important to characterize RSV epidemiology and its impact on healthcare resources as core pandemic prevention strategies have been lifted.

**Figure 1.**
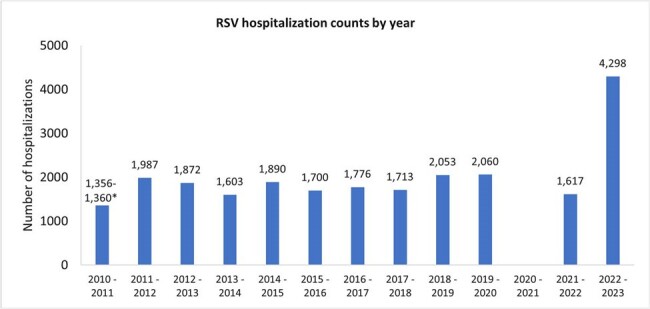
Counts of RSV hospitalizations in Ontario children and adolescents aged ≤17 years across 2010 – 2023. *Exact counts for 2010 – 2011 and 2020 – 2021 were suppressed for privacy reasons.

**Methods:**

Patients aged ≤ 17 years hospitalized with RSV between July 1, 2010 and March 31, 2023 were identified from provincial administrative data at ICES, which captures healthcare encounters within Ontario’s publicly funded healthcare system. Annual outcomes were reported from July 1^st^ to June 30^th^ of the following year.

**Figure 2.**
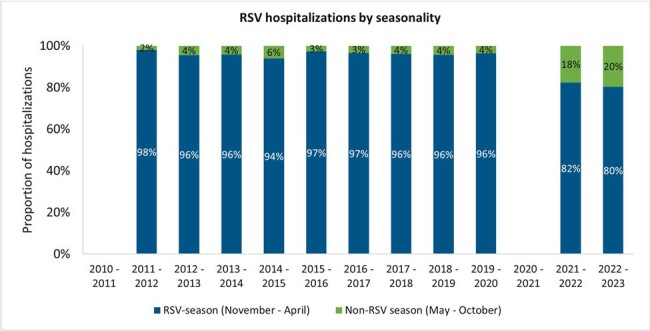
Proportion of RSV hospitalizations in Ontario children and adolescents aged ≤17 years by seasonality across 2010 – 2023. *Exact counts for 2010 – 2011 and 2020 – 2021 were suppressed for privacy reasons.

**Results:**

Between 2010-2020, 1,356-2,060 annual RSV hospitalizations were recorded, with a decrease in 2020-21 season to < 6 cases, followed by an increase to 1,617 (2021-22) and 4,298 (2022-23), likely due to consequences of pandemic-related restrictions (**Figure 1**). Prior to 2020, only 2-6% of RSV hospitalizations occurred during the non-RSV season (May-October); this increased to 18% in 2021-22 and 20% in 2022-23 (**Figure 2**). In 2022-23, hospitalizations were observed to be ∼2-fold that of previous years’ (2010-2021) for those < 12 months, while those hospitalized at 12-< 24 months or 2-17 years old were ∼3-fold and ∼5-7-fold higher, respectively. Interestingly, 2021-22 and 2022-23 had shorter median length of stay (LOS) in the intensive care unit (ICU) even while the same period had the greatest ICU utilization (14-17%; overall study period: 12%). In 2022-23, 55% more children hospitalized at < 4 or 4-< 7 months old had an ICU stay compared to the overall study period while their LOS in ICU remained similar. The median hospitalization costs remained consistent throughout the study period at ∼CAD$5,000, with slightly higher costs observed for 2021-2023 (∼CAD$5,300). Therefore, the total annual cost of RSV hospitalizations more than doubled from ∼CAD$12-16 million during 2010-2022 to ∼CAD$38M in 2022-23 due to the rise in hospitalized cases (**Figure 3**).

**Figure 3.**
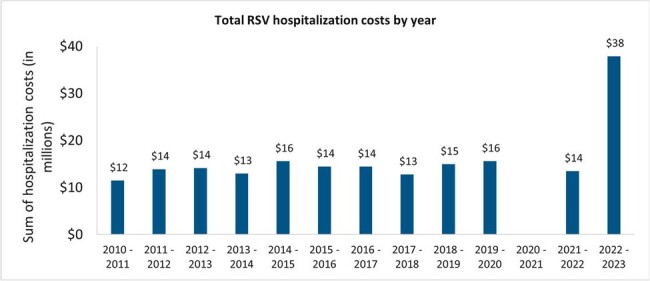
Total costs of RSV hospitalizations in Ontario children and adolescents aged ≤17 years across 2010 – 2023.

There were only <6 RSV hospitalizations reported for 2020 – 2021 likely due to the COVD-19 restrictions. All costs were standardized to 2021 Canadian dollars.

**Conclusion:**

The impact of pandemic measures on RSV hospitalizations were substantial, with most of the consequences observed in 2022-23. Study findings suggest that older children were more impacted by the recent changes in RSV trends, and it is still unclear when pre-pandemic patterns will resume.

**Disclosures:**

**Sazini Nzula, PhD**, Pfizer Canada: Employee **Alexandra Goyette, MSc**, Pfizer: Employee|Pfizer: Stocks/Bonds (Private Company) **Deshayne B. Fell, PhD**, Pfizer Inc.: Employment|Pfizer Inc.: Stocks/Bonds (Private Company) **Ana Gabriela Grajales, MD**, Pfizer Canada ULC: I am currently an employee in Medical Affairs|Pfizer Canada ULC: Stocks/Bonds (Public Company)

